# Aberrant Default Mode Functional Connectivity in Early Onset Schizophrenia

**DOI:** 10.1371/journal.pone.0071061

**Published:** 2013-07-29

**Authors:** Jinsong Tang, Yanhui Liao, Ming Song, Jia-Hong Gao, Bing Zhou, Changlian Tan, Tieqiao Liu, Yanqing Tang, Jindong Chen, Xiaogang Chen

**Affiliations:** 1 Institute of Mental Health, the Second Xiangya Hospital, Central South University, Changsha, Hunan, PR China; 2 Hunan Province Technology Institute of Psychiatry, Changsha, Hunan, PR China; 3 National Laboratory of Pattern Recognition, Institute of Automation, Chinese Academy of Sciences, Beijing, PR China; 4 Department of Radiology, University of Chicago, Chicago, Illinois, United States of America; 5 Department of Radiology, Second Xiangya Hospital, Central South University, Changsha, Hunan, PR China; 6 Department of Psychiatry, First Affiliated Hospital, China Medical University, Shenyang, Liaoning, PR China; 7 The State Key Laboratory of Medical Genetics, Central South University, Changsha, Hunan, PR China; University of Maryland, College Park, United States of America

## Abstract

**Background:**

The default mode network (DMN) has been linked to a number of mental disorders including schizophrenia. However, the abnormal connectivity of DMN in early onset schizophrenia (EOS) has been rarely reported.

**Methods:**

Independent component analysis (ICA) was used to investigate functional connectivity (FC) of the DMN in 32 first-episode adolescents with EOS and 32 age and gender-matched healthy controls.

**Results:**

Compared to healthy controls, patients with EOS showed increased FC between the medial frontal gyrus and other areas of the DMN. Partial correlation analyses showed that the FC of medial frontal gyrus significantly correlated with PANSS-positive symptoms (partial correlation coefficient  = 0.538, Bonferoni corrected P = 0.018).

**Limitations:**

Although the sample size of participants was comparable with most fMRI studies to date, it was still relatively small. Pediatric brains were registered to the MNI adult brain template. However, possible age-specific differences in spatial normalization that arise from registering pediatric brains to the MNI adult brain template may have little effect on fMRI results.

**Conclusion:**

This study provides evidence for functional abnormalities of DMN in first-episode EOS. These abnormalities could be a source of abnormal introspectively-oriented mental actives.

## Introduction

Some brain regions, such as the precuneus, anterior and posterior cingulate cortex (PCC), medial prefrontal cortex (mPFC), parahippocampa, and inferior parietal cortices, are particularly active during “rest” and are deactivated during a variety of cognitive tasks [1], [2]. These brain regions form the “default mode network” (DMN). This concept first emerged in literature in 2001 [3] and has rapidly become a central theme in contemporary cognitive and clinical neuroscience [4]. The DMN is involved in many aspects of brain function, and healthy functional connectivity of the DMN is imperative for normal mental functions. For example, early stage studies suggested that the brain's DMN supports ‘‘self-referential’’ or ‘‘introspective’’ mental activity [5]. Particularly, the mPFC has been linked to internal ‘‘narrative’’ [6], the ‘‘autobiographical’’ self [7], “stimulus independent thought’’ [8] ‘‘mentalizing’’ [9], and ‘‘self-projection’’ [7].

Recently, impairment in the connectivity or activation of the DMN has been linked to many mental disorders. In autism, reduced self-referential, affective, and introspective thought have been revealed to be associated with weak activation of the DMN in the resting state [4]. Reduced deactivation of mPFC and increased deactivation of the PCC are believed to be associated with anxiety disorders [10]. The subgenal cingulate cortex is a prominent region within the DMN and was found to be associated with the length of a depressive episode [11]. Recent studies have demonstrated that functional connectivity of the DMN is disrupted in schizophrenia [12]–[14]. In schizophrenia, positive symptom severity correlated with increased deactivation of the middle frontal regions, precuneus, and the left middle temporal gyrus in an oddball task [1]. Impaired self-monitoring processes and stimulus-independent thought have been associated with abnormally low frequency resting state connectivity [15]. However, all of these studies in schizophrenia are focused on adult onset or mixed onset schizophrenia.

Schizophrenia is characterized by hallucinations or disorganized thinking, loss of goal-directed behaviors, social withdrawal and cognitive deficits [16]. Early-onset schizophrenia (EOS) is defined herein as schizophrenia with onset by 18 years of age [17]. In comparison to patients with adult onset schizophrenia, adolescents with EOS might represent a more homogeneous subgroup associated with greater familial diathesis for the disorder [18]. Therefore, we investigated the functional connectivity of DMN in EOS patients compared with age-matched healthy controls.

## Materials and Methods

### Subjects

42 patients were recruited from the inpatient unit at the Institute of Mental Health at the Second Xiangya Hospital of Central South University. The patients were selected to participate in this study based on the following inclusion criteria: 1) fulfilled the DSM-IV-TR (Diagnostic and Statistical Manual of Mental Disorders, Fourth Edition, Text Revision, American Psychiatric Association, 2000) criteria for schizophrenia, 2) aged 12 to 19, 3) onset of schizophrenia before the 18^th^ birthday, 4) no comorbid Axis I diagnosis, and 5) no mental retardation. Confirmation of the schizophrenia was made by clinical psychiatrists for all patients, using the Structured Clinical Interview for DSM-IV-TR, Patient version (SCID-I/P) [19]. These patients were interviewed six months after the study to review the diagnosis, and all patients received a final diagnosis of schizophrenia. Patients were free of concurrent psychiatric disorders and had no history of major neurological or physical disorders that could lead to an altered mental state. Ten first-episode adolescents with EOS were excluded due to excessive head motion. Therefore, only 32 first-episode adolescents with EOS were included in data analysis. All 32 patients were recruited during an acute psychotic episode, had short duration of illness (mean: 9.8 months; SD: 3.7 months; range: 3–18 months) at the entry of the study, and a mean age of onset of 15.4 years (SD: 1.4 years). Among the 32 patients, 6 patients were not on any medication, while 16 patients were receiving atypical antipsychotic medications at the time of scanning (risperidone [n = 14], clozapine [n = 1], sulpiride [n = 1], or quetiapine [n = 5]). At the time of scanning, the schizophrenia symptoms were rated by trained and experienced psychiatrists using the Positive and Negative Symptom Scale (PANSS) [20].

38 healthy subjects were recruited by advertisement as the control group. Control subjects were free of any known psychiatric condition and had no family history of psychosis in their first-degree relatives. None of the subjects have a past history of major physical or neurological illness. Patients and healthy controls with a current or history of drug use or abuse were excluded from this study. 6 healthy controls were excluded due to excessive head motion, so only 32 healthy controls were finally included in data analysis. All patients and control subjects were right-handed. Patients and control subjects were statistically similar in terms of gender composition, age, educational level, and head motion (see table 1).

**Table 1:** Characteristics of EOS and control groups in fMRI analysis.

	Control (n = 32)	EOS (n = 32)	P value
Age(year)	16.4±0.9	16.2±1.2	0.39^a^
Gender (male/female)	15 M/17 F	16 M/16 F	0.8^ b^
Education(year)	9.7±0.7	9.4±1.5	0.29 a
Onset age(year)	–	15.4±1.2	–
Head motion (translation, mm)	0.46±0.22	0.48±0.25	0.75 a
Head motion (rotation, degree)	0.45±0.26	0.43±0.28	0.75 a
PANSS Positive Symptoms	–	22.4±3.2	–
PANSS Negative Symptoms	–	20.8±3.3	–
PANSS General psychopathology	–	34.4±3.5	–
CPZ equivalent (mg)	–	229.3±188.7	–

a T-test; ^b^Chi-square tests; EOS: Early-onset schizophrenia.

The study protocol was approved by the university ethics committee (The Review Board of Second Xiangya Hospital of Central South University), and the studies were carried out in accordance with the Declaration of Helsinki. Written informed consent was received from all participants and their parents or guardians after the risks and benefits were discussed in detail. If the patient failed to fill out the consent form correctly for more than two times, the parents or guardians were asked to fill out the consent form.

### Imaging data acquisition

Scans were performed on a 1.5 T GE MRI scanner. Foam pads were used to limit head motion and reduce scanner noise. The functional scanning was carried out in the dark, and the participants were explicitly instructed to keep their eyes closed, relax, and to not move during the scan. Functional images were acquired with gradient-echo echo-planar imaging with the following parameters: TR  = 2.0 s, TE  = 40 ms, field of view  = 24 cm, acquisition matrix  = 64×64, flip angle  = 90°, in-plane resolution  = 3.75×3.75 mm, slice thickness  = 5 mm, gap  = 1 mm, 20 slices, axial acquisition, Time point  = 180, scan time  = 6 min. T1 structural image was only acquired from some of the subjects because several subjects could not remain motionless for the duration of the fMRI scan.

### Imaging data preprocessing

Functional MRI data were preprocessed using Statistical Parametric Mapping (SPM5, http://www.fil.ion.ucl.ac.uk/spm/). The first 10 time points from each functional image were discarded to allow for equilibration of the magnetic field. The remaining data were realigned using INRIalign, a motion-correction algorithm unbiased by local signal changes [21]. The participants included in the analysis should have less than 1 mm maximum displacement in the x, y or z direction and less than 1° of angular rotation about each axis. Six healthy controls and 10 EOS patients were excluded from data analysis because they did not achieve the above criteria. Data were then spatially normalized into standard Montreal Neurological Institute space [22], spatially smoothed with a 8×8×8 mm^3^ full width at half-maximum Gaussian kernel. The data (originally acquired at 3.75×3.75×5 mm^3^) were slightly subsampled to 3×3×3 mm^3^, resulting in 61×73×61 voxels.

### Independent component analyses

Independent Component Analyses were preprocessed in 32 first-episode adolescents with EOS and 32 healthy controls. We used the Group ICA fMRI Toolbox (GIFT) to analyze the fMRI data with spatial ICA [23]. We first used a minimum description length algorithm to find the optimal number of spatially independent components [24]. The mean dimension estimation was 28.14 (SD  = 4.07). Therefore, 28 brain components were decomposed from images by GIFT. The data were then further compressed with principal component analysis. Spatial ICA was applied to this reduced data-set and performed on all of the subjects at once with the Infomax algorithm [25]. After discarding the components that were related to artifacts, we used published methods [1], [26] to select components of the default mode network. We compared each component's spatial map with *a priori* map of white matter and cerebral spinal fluid. Components that had a higher correlation with these maps were not considered to be meaningful activations and were discarded. In this study, the component with the highest r value was defined as the most related component. A component with r = 0.176 was considered as a white matter component, while a component with r = 0.198 was considered as a CSF component. We then compared the remaining components with *a priori* map of default mode network provided in GIFT. After this procedure, two components were identified as components of the DMN. The DMN components were then converted to z values for further analysis.

Individual maps of each group were subjected to a random effect one sample t-test in SPM5 and thresholded at p<0.05 to correct for family-wise error and create a group-specific component map. These two maps of EOS patients and healthy control were combined as a mask for group analyses within the DMN component. Thus, results are not biased by DMN maps defined from healthy control or EOS patients only.

### Group comparison

Individual DMN component GIFT maps were entered into SPM5 for group analyses. The z values in these individual maps represent the fit of a specific voxel BOLD time course to the group averaged component's time course. Thus, group analyses test the connectivity strength (i.e. signal synchronization) of each voxel to the whole spatial component. Random effects two-sample t-test examined group difference. The resulting statistical maps were masked with the study-specific general map of the relevant component to explore results within this network only. Age, gender, years of education and antipsychotic treatment were treated as confounding covariates. Clusters of 20 voxels (a cluster size equal to 3×3×3 mm^3^) or greater, surviving a false discovery rate (FDR) with corrected threshold of p<0.05 were considered significant.

### Post-hoc correlation analyses

Correlations between mean Z values of the significant clusters from group analysis and clinical factors (PANSS-positive symptoms, PANSS-negative symptoms, PANSS-general psychopathology, PANSS-total score, age of psychosis onset, and duration of psychosis in patients with EOS) were calculated by partial correlation analysis (P<0.05). Again, analyses were controlled for age, gender, years of education and antipsychotic treatment.

## Results

### Component identification

**Figure 1:**
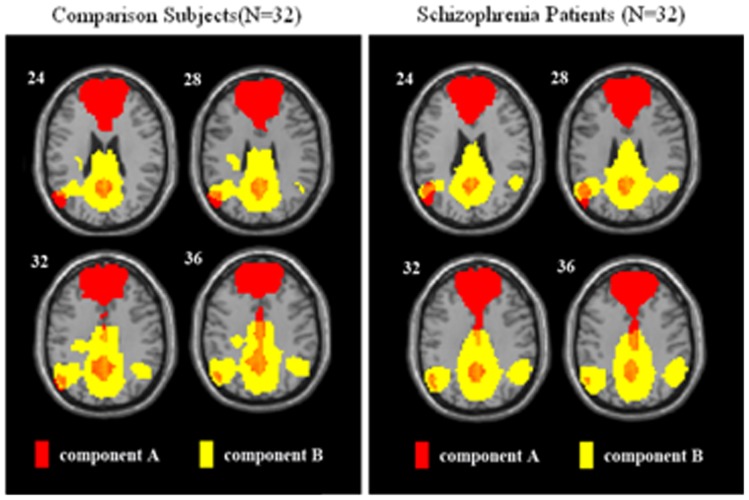
Default Mode Network Map for Patients with Early onset Schizophrenia and Healthy Comparison Subjects.

Independent component analysis yielded 2 components that included brain areas previously reported to be part of the DMN [26] (Fig. 1): (A) anterior component of DMN; (B) posterior component of DMN; Spatial correlation with the DMN a priori mask revealed that these components were the two highest ranked components (r = 0.28 and 0.27, respectively), indicating that regions in these components closely resembled regions in the DMN mask.

### Group differences in DMN connectivity

**Figure 2:**
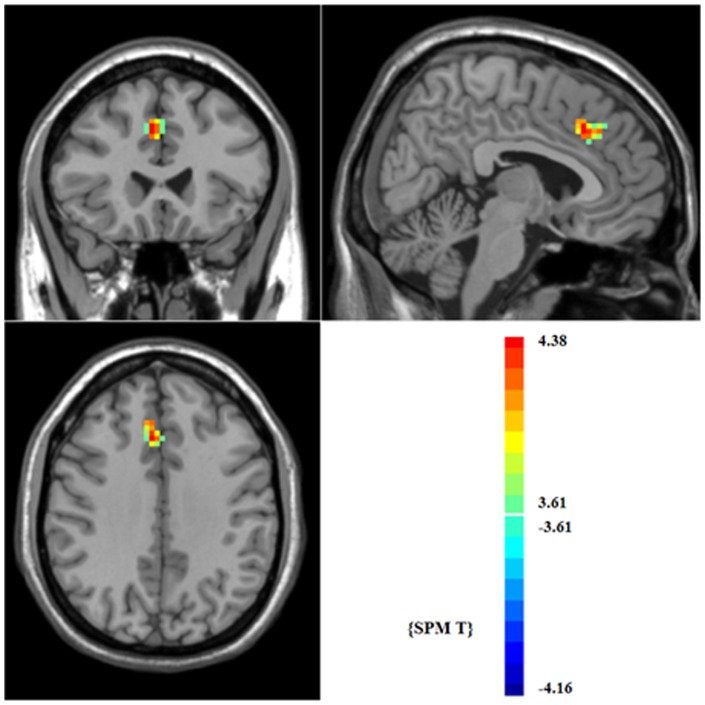
Brain regions with significantly increased strength of connectivity in patients with EOS compared with healthy controls.

Although the DMN maps of patients and controls were similar overall, voxelwise two-sample t-test revealed significant differences in connectivity for component A in the medial frontal gyrus (x = 6, y = 24, z = 36; t = 4.38, FDR corrected P<0.05). EOS patients showed increased strength of connectivity compared to healthy controls (Fig. 2). There were no significant differences in connectivity for component B.

### Relationship between clinical factors and connectivity of medial frontal gyrus

Partial correlation analyses showed that the connectivity of medial frontal gyrus significantly correlated with PANSS-positive symptoms (partial correlation coefficient  = 0.538, Bonferroni corrected P = 0.018) controlled for age, gender, years of education and antipsychotic treatment.

## Discussion

Functional and structural abnormalities in both white and gray matters of EOS patients have been widely reported [27]. For instance, Alexander-Bloch investigated the topology of networks derived from resting-state fMRI and revealed that modularity of brain functional networks was significantly reduced in childhood-onset schizophrenia [28], [29]. On the other hand, impairments in connectivity or activation in the DMN have also been observed in adult onset or mixed onset schizophrenic [4]. In this study, we observed that EOS patients showed a significant increase in strength of connectivity in the medial frontal gyrus (MFG) compared to age-matched healthy controls. The DMN maps identified in this study are consistent with previous findings in non-EOS patients [30]–[32]. Moreover, the connectivity of MFG significantly correlated with PANSS-positive symptoms. Our findings suggest that functional abnormalities in the DMN may be associated with abnormal mental activation in schizophrenia.

Atypical functional connectivity in the DMN has been widely observed in schizophrenic patients. Several studies observed increased resting state functional connectivity within the DMN of schizophrenic patients [10], [33]. However, decreased resting state DMN connectivity has also been observed in schizophrenic patients [16], [34]. For example, a study that examines schizophrenic patients during rest observed disconnectivity of low-frequency oscillatory activity in the PCC, medial prefrontal, lateral and cerebellar regions [16]. Garrity et al observed greater deactivation in the frontal gyrus in the DMN and decreased activity of the ACC in schizophrenic patients relative to healthy controls during an auditory oddball task [1]. Moreover, both increased and decreased connectivity have been observed in a group of schizophrenic patients. For example, Mingoia et al observed decreased pICA-derived connectivity in the right and left dorsolateral prefrontal cortices, bilateral medial frontal cortex, left precuneus and left posterior lateral parietal cortex, but increased connectivity in the right amygdala, left orbitofrontal cortex, right anterior cingulated and bilateral inferior temporal cortices [35]. Besides abnormal connectivity observed within the DMN, changes in connectivity between the DMN and other networks have also been observed. For instance, a recent study using spatial ICA found increased connectivity between the DMN and other resting state networks in schizophrenic patients [36]. In this study, we observed an increased resting state functional connectivity in the medial frontal gyrus of EOS patients. These divergent observations among these studies may be attributed to many factors, including differences in patient population, task instructions, methods of data analysis, statistical approaches, and whether the potential effects of psychotropic medicine were accounted for [34].

A previous study revealed that greater deactivation of the MFG, precuneus, and left MTG is correlated with positive symptoms of schizophrenia [1]. Mingoia et al study reported an inverse correlation of negative symptoms with activity in the right anterior PFC of schizophrenic patients at rest [35]. Importantly, we found that the increased resting state functional connectivity in the MFG significantly correlated with PANSS-positive symptoms in EOS. The DMN is thought to reflect internal, self-referential, and stimulus-independent thought. Therefore, it is not surprising that there are anomalies in this network in patients with EOS. The enhanced connectivity within the DMN may blur the normal boundary between internal thoughts and external perceptions. The failure to recognize internally generated thought is believed to be a fundamental aspect of schizophrenia [37]. Also, hallucination is thought to be the result of a blurring of internal reflection and external perception. In addition, many symptoms of schizophrenia involve an exaggerated sense of self-relevance in the world, such as paranoid ideation. Our and others' studies suggest that altered connectivity within the DMN may have implications for the pathogenesis of schizophrenia.

In this study, pediatric brains were registered to the MNI adult brain template. We acknowledge that registering pediatric brains to the MNI adult brain template could result in some age-specific differences in spatial normalization. However, these differences are unlikely to affect fMRI results because fMRI has a relatively low spatial resolution [29], [38], [39] and functional activity is represented by regional mean time series averaged over multiple voxels comprised of regions of the parcellation template image. In conclusion, the investigation of resting brain function provides novel access into the pathophysiology of schizophrenia. Aberrant functional connectivity of the DMN may explain abnormalities in the coordination of information processing in the brain of EOS patients at rest. The findings in this study suggest the involvement of DMN in schizophrenia.
